# Research on the mechanism of consumer participation in value co-creation by innovative enterprises: An evolutionary game analysis framework

**DOI:** 10.1371/journal.pone.0297475

**Published:** 2024-05-15

**Authors:** Yuhua Liu

**Affiliations:** International School, Malaysia University Business School of Mahsa, Selangor, Malaysia; Southwest University, CHINA

## Abstract

The profound changes brought about by informatization and digitalization have given rise to the user-centered innovation concept, and value co-creation by enterprises has become an inevitable trend. It has become a pressing issue for scholars to analyze the mechanism of consumer participation in the value co-creation of innovative enterprises. In this paper, by establishing an evolutionary game model between consumers and innovative enterprises, we analyze in depth the mechanism of consumer participation in the value co-creation of innovative enterprises. The results show that the initial cooperation probability between consumers and innovative enterprises directly affects their strategic choices; the establishment of reward mechanisms makes consumers more inclined to choose active participation in value co-creation strategies; as the probability of non-cooperation between the two parties being reported increases, the probability of consumers and innovative enterprises choosing cooperation also increases. Studying the mechanism of consumer participation in the value co-creation of innovative enterprises has essential theoretical and practical significance for enterprises to achieve value creation, enhance competitiveness, and promote innovation. This study not only enriches and develops relevant theories but also provides guidance and support for the practice of enterprises, promoting sustainable development and successful co-creation.

## 1. Introduction

Under the background of globalization, digitalization, and social transformation, the business environment faced by enterprises has become increasingly complex and dynamic. On one hand, the development of information and digital technology has imposed stricter requirements on the development of enterprises. Traditional business models and modes can no longer meet the needs of the times. On the other hand, stakeholders’ influence and power over enterprises, including consumers, employees, shareholders, government, social organizations, suppliers, and others, have been enhanced. Among stakeholders, consumers hold a pivotal position. With increased power and decision-making autonomy in purchasing, consumers are no longer passive participants but have become vital forces actively involved in and influencing enterprise decisions.

Currently, value co-creation can be broadly categorized into two main theories. One is the experiential value co-creation theory based on consumer experience, which suggests that through interactions with consumers, enterprises can create personalized experiences [1]. The other is the service-dominant logic of value co-creation, which argues that value is co-created through the collaboration and interaction of multiple stakeholders, including employees, consumers, and enterprises. Stakeholders contribute their knowledge and skills to the value-creation process of enterprises, thus achieving value-creation [2,3]. Both of these theories indicate the vital role consumers play in transitioning from a product-dominant logic to a consumer experience logic and a service-dominant logic. The relationship between consumers and enterprises is a co-creative one aimed at achieving value co-creation, stimulating product and service innovation, and ultimately gaining competitive advantage [4,5]. Therefore, enterprises must actively collaborate with stakeholders, especially consumers, to engage in value co-creation and jointly tackle the challenges and opportunities brought about by societal changes.

Consumers are critical actors in the value-creation ecosystem. Consumers contribute significantly through effective interactions as co-creators of value for enterprises [1,6]. The rise of internet services and social media has provided a favorable platform and channel for consumer participation in value creation for enterprises [7,8]. Consumers can co-create value with enterprises through online and offline channels [9,10]. Enterprises can improve their products and services online by considering consumer reviews and feedback [11]. In offline channels, enterprises provide opportunities for consumers to experience their products and services. This experiential engagement positively influences consumer participation in value co-creation and enhances brand loyalty and satisfaction [12,13]. Furthermore, enhancing enterprises’ brand value also contributes to consumer participation in value co-creation [14]. It is important to note that whether in online or offline channels, paying attention to differentiated consumer demands is essential for value co-creation by enterprises [15].

In value co-creation with consumers, enterprises must focus on guiding consumer psychology. Understanding consumer needs from dimensions such as psychological ownership, self-identity, and spatial requirements can enhance consumer brand loyalty and ultimately improve the competitiveness of enterprises through value co-creation [16].

However, it is undeniable that many factors still hinder consumer participation in value co-creation with enterprises. On the one hand, the asymmetry and inapplicability of resources and information between consumers and enterprises lead to a lower willingness of consumers to participate in value co-creation [17,18]. On the other hand, the lack of consumer expertise results in lower contribution capabilities in the value co-creation process [19]. Additionally, consumer consumption inertia, perceived complexity, perceived risk, and perceived justice can also become critical barriers that hinder consumer participation in value co-creation [20].

Evolutionary game theory mainly studies the dynamic process of subject evolution over time, which is different from traditional game theory’s complete rationality and static analysis. Based on bounded rationality, evolutionary game theory is a dynamic game theory. Due to its ability to help understand and explain the evolution and change of individual and group behavior, as well as the interaction and results of inter-group games, evolutionary game theory has been widely applied in fields such as biology, economics, management, and social behavior.

For instance, evolutionary game theory is used in biology to predict social behavior and other characteristics that influence individual interaction patterns and to analyze the social dominance hierarchy of group-living animals by combining evolutionary game theory with behavioral mechanisms [21].

In economics, scholars have utilized a combination of epidemiological models and behavioral dynamics concepts from evolutionary game theory to analyze the gradual weakening of compliance with economic shutdowns over time and the development of shield immunity during the COVID-19 pandemic [22]. Additionally, researchers have employed evolutionary game theory models to analyze the issue of data openness in the digital economy, focusing on critical actors such as data providers, users, and regulatory agencies [23].

In management, scholars have used evolutionary game theory to study reputation management in the Internet of Vehicles (IoV) [24]. Evolutionary game models have also been employed to analyze group decision-making in signed social networks, specifically examining the dynamics between selfish and collectivist agents [25].

In the field of social behavior, scholars have utilized reputation mechanisms and Markov process-based individual game transitions to describe changes in individual psychology [26]. Evolutionary multigame models combined with dynamic complex networks have been employed to analyze and predict group decision-making behavior in interactive environments [27]. Furthermore, the application of evolutionary game theory has been explored in constructing centralized exclusionary institutions as global exclusion models and analyzing their potential impacts on the replicator dynamics of public goods games [28]. Additionally, numerous reviews have highlighted the wide-ranging applications of evolutionary game theory in both natural and social sciences [29].

Although the literature above analyzes consumer participation in value co-creation with enterprises, some limitations remain. Firstly, current research mainly relies on qualitative and case analysis methods to analyze the process of consumer participation in value co-creation with enterprises. Secondly, the above studies should have considered the issue of consumer strategic choices in the analysis of consumer participation in value co-creation with enterprises. Thirdly, current research has yet to analyze the path changes in consumer participation in value co-creation with enterprises. In comparison to existing literature on value co-creation in innovative enterprises, this study makes several contributions in the following aspects:

1. Currently, scholars mainly rely on case studies and qualitative descriptions to research value co-creation in innovative enterprises, with few scholars conducting in-depth analyses using empirical methods or mathematical models. This study takes consumers and innovative enterprises as the game participants. It conducts a comprehensive mathematical analysis of consumer participation in value co-creation with innovative enterprises by establishing an evolutionary game model.
2. In analyzing the process of consumer participation in value co-creation with innovative enterprises, this study considers the issue of consumer strategic choices, which breaks the existing research paradigm of value co-creation in innovative enterprises. It expands the research boundaries and enriches the research content of value co-creation in innovative enterprises.
3. After using the evolutionary game model to analyze the process of consumer participation in value co-creation with innovative enterprises, this study further simulates and analyzes the evolutionary path of consumer participation in value co-creation with innovative enterprises using Matlab. It also investigates the impact of parameter changes on value co-creation in innovative enterprises.

The remaining structure of this article is arranged as follows: Part 2 analyzes the underlying mechanisms of consumer participation in value co-creation with innovative enterprises. Part 3 proposes research hypotheses for consumer participation in value co-creation with innovative enterprises. Part 4 establishes an evolutionary game model with consumers and innovative enterprises as the primary entities. Part 5 uses Matlab to simulate the evolutionary path of consumer participation in value co-creation with innovative enterprises. Part 6 presents the conclusions and discussions of this study.

## 2. Analysis of mechanisms

**Fig 1:**
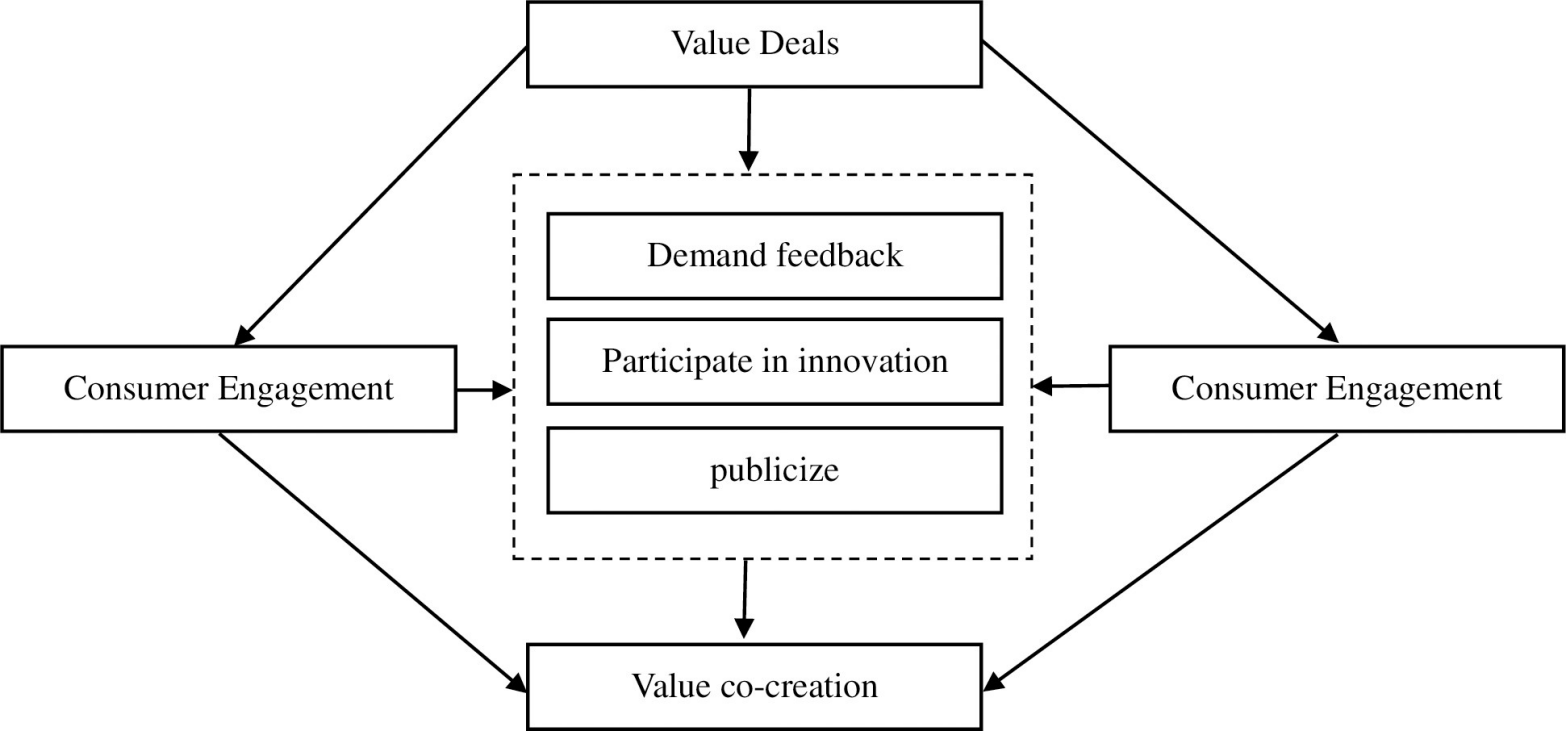


With the rapid development of information technology, represented by digital technology, innovative enterprises must continuously update their technology and business models to adapt to technological advancements. Engaging in value co-creation enables enterprises to leverage new technologies better and provide more advanced products and services to meet consumer needs. Consumer participation in value co-creation with innovative enterprises benefits both parties. On one hand, it enhances consumer trust and loyalty towards the enterprise. On the other hand, it provides the enterprise with diverse, innovative ideas and creativity. Consumers can contribute novel insights and perspectives, challenging the existing mindset of the enterprise and driving innovation and improvement in products and services. Consumer participation also helps enterprises better identify product quality issues and provides directions and opinions for improvement. Fig 1 depicts the logic and mechanisms of consumer participation in value co-creation with innovative enterprises.

From Fig 1, it can be observed that when consumers participate in the value co-creation process with innovative enterprises, they go through the following specific stages. The first stage is feedback on consumer needs. Through consumer communication and feedback, innovative enterprises can better understand market demands, make timely adjustments to their products or services, and enhance their competitiveness. The second stage is consumer involvement in innovation. As participants in the innovation process, consumers can provide new ideas and insights, fostering innovation and development within the enterprise. Innovative enterprises attract consumer participation through innovation and social activities, collaborating to create value. The third stage is consumer word-of-mouth promotion. As loyal users of the enterprise, consumers can promote the products and services to others through word-of-mouth, helping expand the market. The innovative enterprise earns consumer trust and support by delivering high-quality products and services, thus driving its growth.

Behind the mechanisms of consumer participation in value co-creation with innovative enterprises, the enterprise must establish an open, transparent, and interactive cultural atmosphere that encourages consumer involvement in innovation and value co-creation. The enterprise must also establish corresponding participation platforms and tools that enable consumers to interact and engage efficiently. By collaborating with consumers, the enterprise can better meet market demands, increase the success rate of innovation, and enhance market competitiveness.

## 3. Model Hypothesis

The involvement of consumers in co-creating value with innovative enterprises has been a topic of significant academic interest. As one of the most important stakeholders in this process, consumers are critical in co-creating value with innovative enterprises. In order to further analyze the underlying mechanisms of consumer participation in value co-creation with innovative enterprises, as well as the strategic choices made by both parties, this paper proposes the following hypotheses.

Hypothesis 1: In the value co-creation process of innovative enterprises, this study assumes the presence of only two game entities. On the one hand, there is the innovative enterprise, which serves as the primary carrier of value co-creation and acts as a central force in the value co-creation process. On the other hand, there are the consumers, who are the primary participants in value co-creation with the innovative enterprise and play a crucial role in enterprise value co-creation.
Hypothesis 2: Although innovative enterprises (*E*) and consumers (*C*) are essential participants in value co-creation, both parties are characterized by bounded rationality. Due to differences in cognitive abilities, information acquisition, time constraints, and experience, innovative enterprises (*E*) and consumers (*C*) cannot make optimal decisions based on unlimited information and analysis. Instead, they have to make decisions based on limited time and information. Moreover, the decision-making process of both parties is also influenced by factors such as emotions, biases, habits, and risk aversion when making choices within limited information. Therefore, innovative enterprises (*E*) and consumers (*C*) seek to maximize their interests in the game process. They also adjust their strategies based on changes in their benefits and the strategies of the other party in order to achieve maximum self-interest.
Hypothesis 3: To simplify the subsequent analysis, this study assumes that the innovative enterprise (*E*) has only two strategies in the game process. One strategy is active value co-creation, while the other is passive. Therefore, the strategy space of the innovative enterprise (*E*) can be represented as = (active value co-creation, passive value co-creation). Simultaneously, it is assumed that the consumers () have only two strategies. One strategy is active participation in value co-creation, while the other is passive participation. The strategy space of the consumers (*C*) can be represented as = (active participation in value co-creation, passive participation in value co-creation). Additionally, it is assumed that the innovative enterprise (*E*) has a probability of choosing the active value co-creation strategy as *x* and a probability of choosing the passive value co-creation strategy as 1 − *x*. Furthermore, it is assumed that the consumers (*C*) have a probability of choosing active participation in the value co-creation strategy as *y* and a probability of choosing passive participation in the value co-creation strategy as 1 − *y*. Both *x* and *y* are probabilistic variables and *x*,*y* ∈ [0,1]. Additionally, both variables are functions of time *t*.
Hypothesis 4: When the innovative enterprise chooses the active value co-creation strategy, it invests significant human, material, and financial resources—for example, research and development costs. Innovative enterprises need to invest resources in research and development to create new products or enhance the value of existing products. This may involve costs such as human resources for research and development teams, equipment, and technology inputs. Communication and coordination costs. When engaging in value co-creation with consumers, innovative entrepreneurs need to engage in more communication and coordination efforts. This may include communicating with consumers, gathering feedback, addressing questions, coordinating needs and expectations, etc. These communication and coordination efforts require time, workforce, and resource investments. Let us assume the cost of these activities as *c*_*E*1_. Similarly, when the innovative enterprise chooses the passive value co-creation strategy, it incurs certain costs. For instance, if the innovative enterprise fails to listen to consumers’ feedback and address their issues, it may negatively impact the enterprise’s reputation. This may result in customer attrition due to product and service problems. Let us assume the cost of this scenario as *c*_*E*2_. Innovative enterprises obtain additional benefits when they successfully engage in value co-creation. For instance, consumer loyalty increases. Innovative entrepreneurs can establish deeper relationships and enhance consumer loyalty through proactive interactions and consumer participation—product and service improvements. Innovative entrepreneurs better understand consumer needs and preferences by involving consumers in the innovation and improvement of products and services. The feedback and suggestions consumers provide during the co-creation process can help identify shortcomings in products and services and drive corresponding improvements. Let us assume the value of these additional benefits is denoted as *R*_*E*1_.
Hypothesis 5: When consumers choose active participation in a value co-creation strategy, they incur certain costs. For example, time costs. Consumers actively participating in value co-creation strategies may require more time to engage in activities, provide feedback and suggestions, communicate and collaborate with the firm, etc. These time costs may consume consumers’ leisure time or working hours—energy costs. Participating in value co-creation strategies requires consumers to invest more energy in thinking and providing valuable opinions, participating in discussions and feedback, and so on. This may require them to gain a deeper understanding of the product and the market, research and analyze relevant issues, and engage in continuous learning and reflection. Let us assume the cost of these activities as *c*_*C*_.

Moreover, when consumers actively participate in value co-creation strategies, they are rewarded with economic incentives. By providing valuable feedback, suggestions, and opinions, consumers actively contribute to improving products and services. In recognition of their participation, firms may provide economic incentives to these contributors, such as coupons, discounts, or gift cards. Let us assume the value of these incentives is denoted as *ω*.

Consumers may face certain losses when they passively participate in value co-creation strategies. For instance, consumers may need more personalized customized products or services. If consumers opt for passive participation, they may not be able to enjoy tailored products or services, instead having to settle for generic offerings that may only partially meet their needs. Consumers may also miss opportunities for interaction and engagement with innovative firms. By choosing passive participation, they may forego these opportunities for interaction and engagement, thereby missing the chance to contribute their creativity and insights. Additionally, consumers may miss out on opportunities for interaction and sharing with other consumers. In value co-creation, consumers can interact and communicate with each other through sharing experiences, recommending products, and more. This interaction and sharing can help consumers gain more information and knowledge, enabling them to make more informed decisions. By opting for passive participation, consumers may miss out on these opportunities for interaction and sharing, potentially needing more information and knowledge to make fully informed decisions. Let us assume the value of these losses is denoted as *f*.

The probability of both parties being detected as non-cooperative and reported is represented as *η*. Reporting non-cooperative behavior can prompt both parties in a game to reassess their actions and strengthen adherence to cooperative norms. Reporting can serve as a warning and deterrent, making participants realize the adverse consequences of non-cooperative behavior, guiding them to comply with cooperative rules, and ultimately achieving value co-creation. However, the success of reporting depends on several factors, such as the rigor of regulatory agencies, the number of reporters, and the effectiveness of the reporting system. Therefore, let us denote the probability of successful reporting as *η*.

Hypothesis 6: Even if consumers and innovative enterprises choose not to participate in the value co-creation game, both parties still receive fundamental benefits. Let us assume that the fundamental benefit for the innovative enterprise is denoted as *R*_*E*_, and the consumer’s fundamental benefit is denoted as *R*_*C*_.

Table 1 shows the variable parameters and their meanings for consumers and innovative enterprises, with detailed content in Table 1.

**Table 1:** Variables and their meanings for game participants.

Variables	Meanings of variables
*c*_*E*1_	Cost for innovative enterprises to actively engage in value co-creation
*c*_*E*2_	Cost for innovative enterprises to passively engage in value co-creation
*R*_*E*1_	Profit for innovative enterprises after successful value co-creation
*c*_*C*_	Cost for consumers to actively participate in value co-creation
*ω*	Rewards for consumers
*f*	Losses faced by consumers
*Η*	The probability of both sides of the game being reported when not cooperating
*R*_*E*_	The fundamental benefits of innovative enterprises
*R*_*C*_	The fundamental benefits of consumers

The payoff matrix for the evolutionary game between consumers and innovative enterprises can be presented based on the above assumptions. For detailed information, please refer to Table 2.

**Table 2:** Payoff matrix for the game between consumers and innovative enterprises.

		Consumers(*C*)	
		Actively participating in value co-creation *y*	Passively participating in value co-creation 1 − *y*
Innovative enterprises(*E*)	Proactively engaging in value co-creation *x*	*R*_*E*_ − *c*_*E*1_ *R*_*C*_ + *ω* − *c*_*C*_	*R*_*E*_ + *R*_*E*1_ − *c*_*E*1_ *R*_*C*_ − *f* − *c*_*C*_
	Passively engaging in value co-creation 1 − *x*	*η*(*R*_*E*_ − *c*_*E*2_) *η*(*R*_*C*_ + *ω* − *c*_*C*_) + (1 − *η*)(*R*_*C*_ + *ω*)	*η*[(*R*_*E*_ + *R*_*E*1_ − *c*_*E2*_) + (*R*_*E*_ − *c*_*E2*_)] *η*(*R*_*C*_ − *f* − *c*_*C*_) + (1 − *η*)*R*_*C*_

## 4. Analysis of the co-creation of value between innovative enterprises and consumers

### 4.1 Model establishment and solution

The expected payoffs of consumer and innovative enterprises can be obtained based on the game theory payoff matrix. Assuming the expected payoffs of consumers who actively or passively participate in value co-creation are *u*_*C*1_ and *u*_*C*2_, respectively, the average expected payoff of consumers is ${\overset{-}{u}}_{C}=yu_{C1}+(1-y)u_{C2}$. The specific expressions of expected payoffs are shown in formulas (1)–(3).


$$\begin{array}{l} u_{C1}=x(R_{C}+\omega -c_{C})\\ +(1-x)\{\eta (R_{C}+\omega -c_{C})+(1-\eta )(R_{C}+\omega )\} \end{array}$$ (1)

$$\begin{array}{l} u_{C2}=x(R_{C}-f-c_{C})\\ +(1-x)\{\eta [R_{C}-f-c_{C}]+(1-\eta )R_{C}\} \end{array}$$ (2)

$$\begin{array}{l} {\bar{u}}_{C}=y\{x(R_{C}+\omega -c_{C})+(1-x)[\eta (R_{C}+\omega -c_{C})+\\ \left(1-\eta )(R_{C}+\omega )]\}+(1-y)\{[x(R_{C}-f-c_{C})\right]\\ +(1-x)(\eta (R_{C}-f-c_{C})+(1-\eta )R_{C})\} \end{array}$$ (3)

Similarly, assuming that the innovative enterprise chooses to actively or passively participate in value co-creation and the expected payoffs are *u*_*E*1_ and *u*_*E*2_, respectively, the average expected payoff of the innovative enterprise is ${\overset{-}{u}}_{E}=xu_{E1}+(1-x)u_{E2}$. The specific expressions of the payoffs are shown in formulas (4)–(6).


$$u_{E1}=y(R_{E}-c_{E1})+(1-y)(R_{E}+R_{E1}-c_{E1})$$ (4)

$$u_{E2}=y[\eta (R_{E}-c_{E2})]+(1-y)[\eta [(R_{E}+R_{E1}-c_{E2})+(R_{E}-c_{E2})]]$$ (5)

$$\begin{array}{l} {\overset{-}{u}}_{E}=x[y(R_{E}-c_{E1})+(1-y)(R_{E}+R_{E1}-c_{E1})]\\ +(1-x)[y[\eta (R_{E}-c_{E2})]+(1-y)[\eta [(R_{E}+R_{E1}-c_{E2})+(R_{E}-c_{E2})]]] \end{array}$$ (6)

Based on the principle of Malthusian dynamic equations and formulas (1)–(6), the replicator dynamic equations for consumers and innovative enterprises can be derived. The specific formulas are shown as (7) and (8).


$$\frac{dy}{dt}=y(u_{C1}-\bar{u_{C}})=y(1-y)[xf(1-\eta )+\eta f+\omega ]$$ (7)

$$\begin{array}{l} \frac{dx}{dt}=x(u_{E1}-{\bar{u}}_{E})=\\ x(x-1)[y(R_{E1}+\eta c_{E2}-\eta R_{E}-\eta R_{E1})+(2\eta -1)R_{E}+(R_{E1}-2c_{E2})\eta +c_{E1}-R_{E1}] \end{array}$$ (8)

A two-dimensional dynamical system can be constructed based on the replicator dynamic equations for consumers and innovative enterprises. By solving Eqs (7) and (8), the equilibrium points of the two-dimensional dynamical system can be obtained. After solving the replicator dynamic equations, the equilibrium point obtained is denoted as *E*_1_ (0,0), *E*_2_ (1,0), *E*_3_ (0,1), *E*_4_ (1,1), *E*_5_ (x*, y*).

### 4.2 Stability analysis

To further analyze the stability of consumers and innovative firms at various equilibrium points, it is necessary to solve for the Jacobian matrix of the two-dimensional dynamic system. The Jacobian matrix is derived from the replicator dynamics equations of consumers and innovative firms, taking partial derivatives with concerning the replicator dynamics equations of consumers and innovative firms, as shown in Eq (9) in the following:

$$J_{e}=\left[\begin{array}{ll} \partial F(x)/\partial x & \partial F(x)/\partial y\\ \partial F(y)/\partial x & \partial F(y)/\partial y \end{array}\right]$$ (9)

Based on Eq (9) and the replicator dynamics equations of consumers and innovative firms, the Jacobian matrix of the game system can be calculated as shown in Eq (10).


$$J_{e}=\left[\begin{array}{l} \left(2x-1)[y(R_{E1}+\eta c_{E2}-\eta R_{E}-\eta R_{E1}\right)\\ +(2\eta -1)R_{E}+(R_{E1}-2c_{E2})\eta +c_{E1}-R_{E1}]\\ y(1-y)(1-\eta )f \end{array}\right.\left.\begin{array}{l} x(x-1)(R_{E1}+\eta c_{E2}\\ -\eta R_{E}-\eta R_{E1})\\ \left(1-2y)[x(1-\eta )f+\omega \right] \end{array}\right]$$ (10)

To facilitate further analysis, it is necessary to calculate the determinant(det*J*_*e*_) and trace(*trJ*_*e*_) of the aforementioned Jacobian matrix. The specific expressions are given by Eqs (11) and (12).


$$\begin{array}{l} \text{det}J_{e}=(2x-1)[y(R_{E1}+\eta c_{E2}-\eta R_{E}-\eta R_{E1})+\\ \left(2\eta -1)R_{E}+(R_{E1}-2c_{E2})\eta +c_{E1}-R_{E1}\right]\\ {}*(1-2y)[x(1-\eta )f+\omega ]-\\ x(x-1)(R_{E1}+\eta c_{E2}-\eta R_{E}-\eta R_{E1})*y(1-y)(1-\eta )f \end{array}$$ (11)

$$\begin{array}{l} trJ_{e}=(2x-1)[y(R_{E1}+\eta c_{E2}-\eta R_{E}-\eta R_{E1})\\ +(2\eta -1)R_{E}+(R_{E1}-2c_{E2})\eta +c_{E1}-R_{E1}]\\ +(1-2y)[x(1-\eta )f+\omega ] \end{array}$$ (12)

In order to calculate the eigenvalues of the Jacobian matrix, it is necessary to substitute the equilibrium points obtained from solving the replicator dynamic equations into the Jacobian matrix. The specific details are described in Table 3.

**Table 3:** Eigenvalues of the Jacobian matrix.

Equilibrium point	Eigenvalue	
	*λ*_1_	*λ*_2_
*E*_1_ (0,0)	*ω*	(1 − 2*η*)*R*_*E*_ + (1 − *η*)*R*_*E*1_ + 2*ηc*_*E*2_ − *c*_*E*1_
*E*_2_ (1,0)	*f* + *ω* − *ηf*	−[(1 − 2*η*)*R*_*E*_ + (1 − *η*)*R*_*E*1_ + 2*ηc*_*E*2_ − *c*_*E*1_]
*E*_3_ (0,1)	−*ω*	(1 − *η*)*R*_*E*_ + *ηc*_*E*2_ − *c*_*E*1_
*E*_4_ (1,1)	−(*f* + *ω* − *ηf*)	−((1 − *η*)*R*_*E*_ + *ηc*_*E*2_ − *c*_*E*1_)
*E*_5_ (x*, y*)	*X*	*Y*

Due to the complexity and difficulty in determining the eigenvalues of equilibrium point *E*_5_ (x*, y*) from its formula, this article only analyzes equilibrium points *E*_1_ − *E*_4_. It determines the stability of the equilibrium point based on the signs of the determinant (det*J*_*e*_) and trace (*trJ*_*e*_). Since it is difficult to determine the sign of the eigenvalues, it is necessary to analyze different cases. The specific analysis is shown below.

Scenario 1: When (1 − 2*η*)*R*_*E*_ + (1 − *η*)*R*_*E*1_ + 2*ηc*_*E*2_ − *c*_*E*1_ > 0 and (1 − *η*)*R*_*E*_ + *ηc*_*E*2_ − *c*_*E*1_ > 0, the sign of the eigenvalues can be determined. Based on the sign of the eigenvalues, the sign of the determinant (det*J*_*e*_) and trace (*trJ*_*e*_) can be determined, and the stability of the equilibrium point can be obtained. Please refer to Table 4 for specific details.

Scenario 2: When (1 − 2*η*)*R*_*E*_ + (1 − *η*)*R*_*E*1_ + 2*ηc*_*E*2_ − *c*_*E*1_ > 0 and (1 − *η*)*R*_*E*_ + *ηc*_*E*2_ − *c*_*E*1_ < 0, the sign of the eigenvalues can be determined, and based on the sign of the eigenvalues, the sign of the determinant (det*J*_*e*_) and trace (*trJ*_*e*_) can be determined, which allows for the determination of the stability of the equilibrium point. Please refer to Table 4 for specific details.

Scenario 3: When (1 − 2*η*)*R*_*E*_ + (1 − *η*)*R*_*E*1_ + 2*ηc*_*E*2_ − *c*_*E*1_ < 0 and (1 − *η*)*R*_*E*_ + *ηc*_*E*2_ − *c*_*E*1_ > 0, the sign of the eigenvalues can be determined, and based on the sign of the eigenvalues, the sign of the determinant (det*J*_*e*_) and trace (*trJ*_*e*_) can be determined, which allows for the determination of the stability of the equilibrium point. Please refer to Table 4 for specific details.

Scenario 4: When (1 − 2*η*)*R*_*E*_ + (1 − *η*)*R*_*E*1_ + 2*ηc*_*E*2_ − *c*_*E*1_ < 0 and (1 − *η*)*R*_*E*_ + *ηc*_*E*2_ − *c*_*E*1_ < 0, the sign of the eigenvalues can be determined, and based on the sign of the eigenvalues, the sign of the determinant (det*J*_*e*_) and trace (*trJ*_*e*_) can be determined, which allows for the determination of the stability of the equilibrium point. Please refer to Table 4 for specific details.

**Table 4:** Local equilibrium and stability analysis.

Scenarios	Equilibrium point	*λ*_1_	*λ*_2_	det*J*_*e*_	*trJ*_*e*_	Stability
Scenario 1	(0, 0)	+	+	+	+	Unstable
	(1, 0)	+	-	-	Uncertainty	Saddle point
	(0, 1)	-	+	-	Uncertainty	Saddle point
	(1, 1)	-	-	+	-	ESS
Scenario 2	(0, 0)	+	+	+	+	Unstable
	(1, 0)	+	-	-	Uncertainty	Saddle point
	(0, 1)	-	-	+	-	ESS
	(1, 1)	-	+	-	Uncertainty	Saddle point
Scenario 3	(0, 0)	+	-	-	Uncertainty	Saddle point
	(1, 0)	+	+	+	+	Unstable
	(0, 1)	-	+	-	Uncertainty	Saddle point
	(1, 1)	-	-	+	-	ESS
Scenario 4	(0, 0)	+	-	-	Uncertainty	Saddle point
	(1, 0)	+	+	+	+	Unstable
	(0, 1)	-	-	+	-	ESS
	(1, 1)	-	+	-	Uncertainty	Saddle point

Table 4 shows that in scenario 1, When (1 − 2*η*)*R*_*E*_ + (1 − *η*)*R*_*E*1_ + 2*ηc*_*E*2_ − *c*_*E*1_ > 0 and (1 − *η*)*R*_*E*_ + *ηc*_*E*2_ − *c*_*E*1_ > 0, the game system converges to *E*_4_ (1,1). In this case, consumers are more inclined to choose active participation in the value co-creation strategy. At the same time, the innovative enterprise is also more inclined to choose the active value co-creation strategy. In scenario 2, the game system converges to *E*_3_ (0,1) when (1 − 2*η*)*R*_*E*_ + (1 − *η*)*R*_*E*1_ + 2*ηc*_*E*2_ − *c*_*E*1_ > 0 and (1 − *η*)*R*_*E*_ + *ηc*_*E*2_ − *c*_*E*1_ < 0. Consumers are inclined to choose active participation in the value co-creation strategy, while the innovative enterprise chooses the passive value co-creation strategy. In scenario 3, When (1 − 2*η*)*R*_*E*_ + (1 − *η*)*R*_*E*1_ + 2*ηc*_*E*2_ − *c*_*E*1_ < 0 and (1 − *η*)*R*_*E*_ + *ηc*_*E*2_ − *c*_*E*1_ > 0, the game system converges to *E*_4_ (1,1). In this case, consumers are more inclined to choose active participation in a value co-creation strategy. The innovative enterprise is also more inclined to choose the active value co-creation strategy. In scenario 4, the game system converges to *E*_3_ (0,1) when (1 − 2*η*)*R*_*E*_ + (1 − *η*)*R*_*E*1_ + 2*ηc*_*E*2_ − *c*_*E*1_ < 0 and (1 − *η*)*R*_*E*_ + *ηc*_*E*2_ − *c*_*E*1_ < 0. In this case, consumers are inclined to choose active participation in the value co-creation strategy, while the innovative enterprise is inclined to choose the passive value co-creation strategy. Please refer to Table 4 for more details.

In addition, it is necessary to explain why the equilibrium points do not converge to *E*_1_ (0,0) and *E*_2_ (1,0). Converting equilibrium points to a specific value requires fulfilling certain conditions: det*J*_*e*_ > 0 and *trJ*_*e*_ < 0. Based on the previous assumptions and the eigenvalues of the Jacobian matrix in Table 3, it is impossible to determine the signs of (1 − 2*η*)*R*_*E*_ + (1 − *η*)*R*_*E*1_ + 2*ηc*_*E*2_ − *c*_*E*1_ and (1 − *η*)*R*_*E*_ + *ηc*_*E*2_ − *c*_*E*1_ in the eigenvalues. Therefore, discussing the positive or negative nature of these two eigenvalues in different scenarios is necessary. In all four scenarios mentioned above, regardless of the signs of (1 − 2*η*)*R*_*E*_ + (1 − *η*)*R*_*E*1_ + 2*ηc*_*E*2_ − *c*_*E*1_ and (1 − *η*)*R*_*E*_ + *ηc*_*E*2_ − *c*_*E*1_, it is observed that neither *E*_1_ (0,0) nor *E*_2_ (1,0) satisfies the conditions det*J*_*e*_ > 0 and *trJ*_*e*_ < 0. Therefore, the equilibrium points will not converge to *E*_1_ (0,0) and *E*_2_ (1,0). The eigenvalues *X* and *Y* corresponding to point *E*_5_ (x*, y*) are purely imaginary roots. According to the literature lemma and theorem[30,31], it is known that point *E*_5_ (x*, y*) is a stable equilibrium point but not asymptotically stable. The system’s trajectory will form a closed-loop motion around *E*_5_ (x*, y*). Therefore, the equilibrium points of the system will not converge to *E*_5_ (x*, y*).

## 5. Evolutionary simulation study

Section 4 uses an evolutionary game model to analyze the intrinsic mechanism of consumer participation in value co-creation with innovative enterprises. In this section, numerical simulation analysis of the above results is conducted using Matlab, and the effects of various parameter changes on the evolution path of both sides of the game are further analyzed. The specific analysis is shown below.

### 5.1 Simulation analysis of game results

(1) When (1 − 2*η*)*R*_*E*_ + (1 − *η*)*R*_*E*1_ + 2*ηc*_*E*2_ − *c*_*E*1_ > 0 and (1 − *η*)*R*_*E*_ + *ηc*_*E*2_ − *c*_*E*1_ > 0 hold, the parameters are set as indicated in the second row of Table 5. When (1 − 2*η*)*R*_*E*_ + (1 − *η*)*R*_*E*1_ + 2*ηc*_*E*2_ − *c*_*E*1_ > 0 and (1 − *η*)*R*_*E*_ + *ηc*_*E*2_ − *c*_*E*1_ < 0 hold, the parameters are set as indicated in the third row of Table 5. When (1 − 2*η*)*R*_*E*_ + (1 − *η*)*R*_*E*1_ + 2*ηc*_*E*2_ − *c*_*E*1_ < 0 and (1 − *η*)*R*_*E*_ + *ηc*_*E*2_ − *c*_*E*1_ > 0 hold, the parameters are set as indicated in the fourth row of Table 5. When (1 − 2*η*)*R*_*E*_ + (1 − *η*)*R*_*E*1_ + 2*ηc*_*E*2_ − *c*_*E*1_ < 0 and (1 − *η*)*R*_*E*_ + *ηc*_*E*2_ − *c*_*E*1_ < 0 hold, the parameters are set as indicated in the fifth row of Table 5. For specific details, please refer to Table 5.

**Table 5:** Parameter settings for Scenarios 1–4.

Parameter	*c*_*E*1_	*c*_*E*2_	*R*_*E*1_	*c*_*C*_	*ω*	*f*	*η*	*R*_*E*_	*R*_*C*_
Scenario 1	5	10	10	4	5	6	0.3	5	5
Scenario 2	7	10	10	4	5	6	0.3	5	5
Scenario 3	3	4	3	4	5	6	0.7	10	5
Scenario 4	5	4	3	4	5	6	0.7	5	5

**Fig 2:**
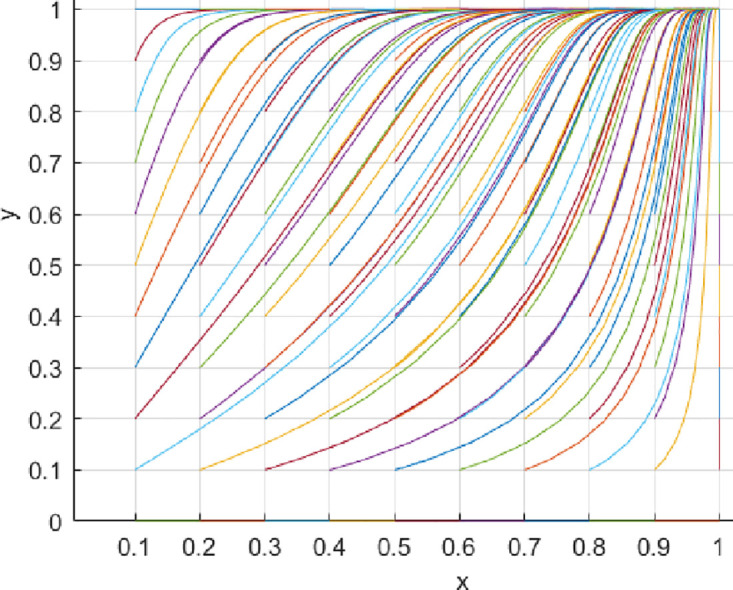


**Fig 3:**
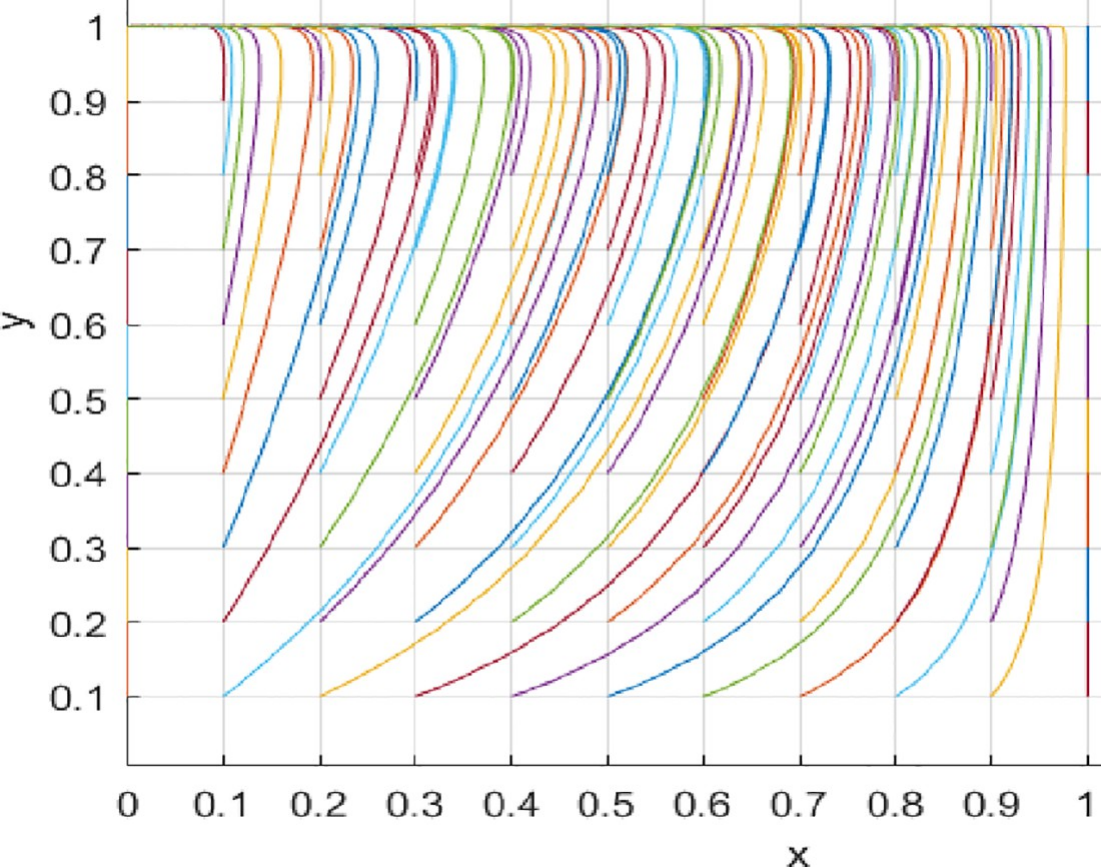


**Fig 4:**
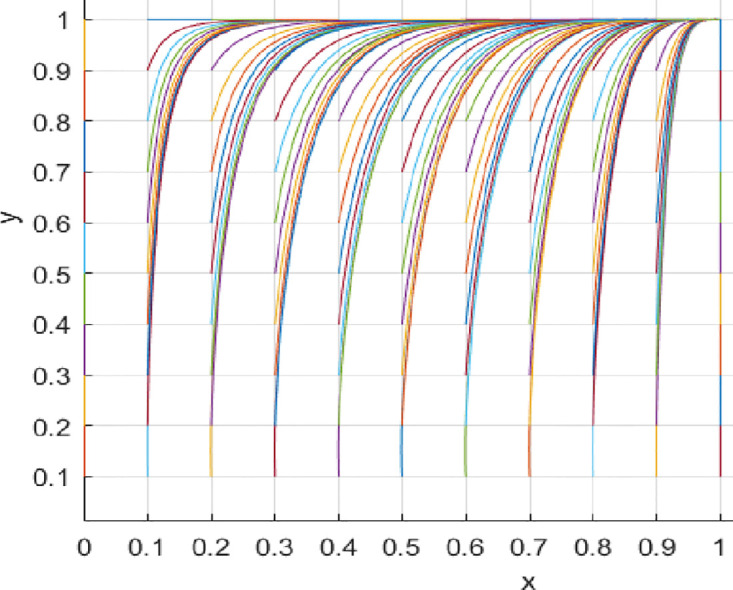


**Fig 5:**
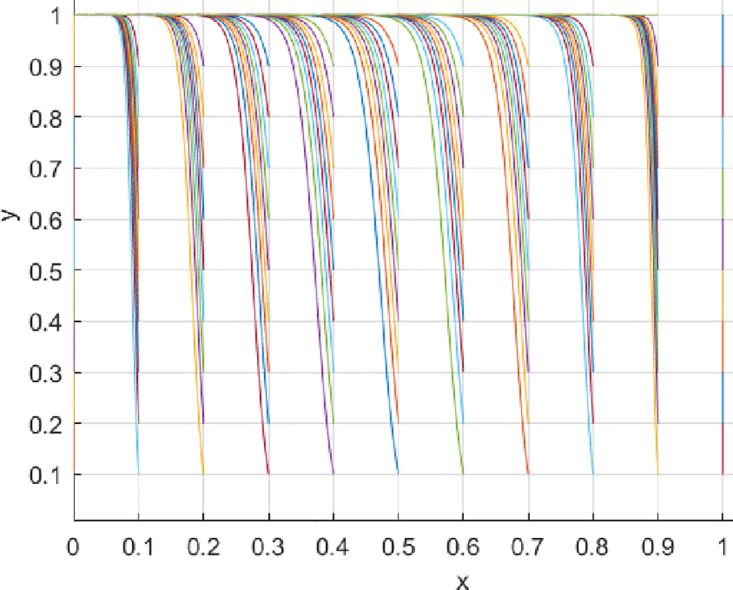


Under the parameter settings for Scenario 1, the game system will converge to point *E*_4_ (1,1). At this point, consumers are more likely to choose a proactive value co-creation strategy, and innovation-oriented enterprises are also more likely to choose to engage in value co-creation proactively. See Fig 2 for more details. Under the parameter settings for Scenario 2, the game system will converge to point *E*_3_ (0,1). At this point, consumers tend to choose a proactive value co-creation strategy, while innovation-oriented enterprises tend to choose a passive value co-creation strategy. See Fig 3 for more details. Under the parameter settings for Scenario 3, the game system will converge to point *E*_4_ (1,1). At this point, consumers are more likely to choose a proactive value co-creation strategy, and innovation-oriented enterprises are also more likely to choose to engage in value co-creation proactively. See Fig 4 for more details. Under the parameter settings for Scenario 4, the game system will converge to point *E*_3_ (0,1). At this point, consumers tend to choose a proactive value co-creation strategy. In contrast, innovation-oriented enterprises tend to choose a passive value co-creation strategy. See Fig 5 for more details.

### 5.2 Simulation analysis of parameter variation

Numerical simulation analysis was performed using Matlab to analyze further the impact of parameter variations on the evolutionary paths of both players in the game. Please refer to the following sections for detailed information.

(1) Variation in Initial Cooperation Probability

**Fig 6:**
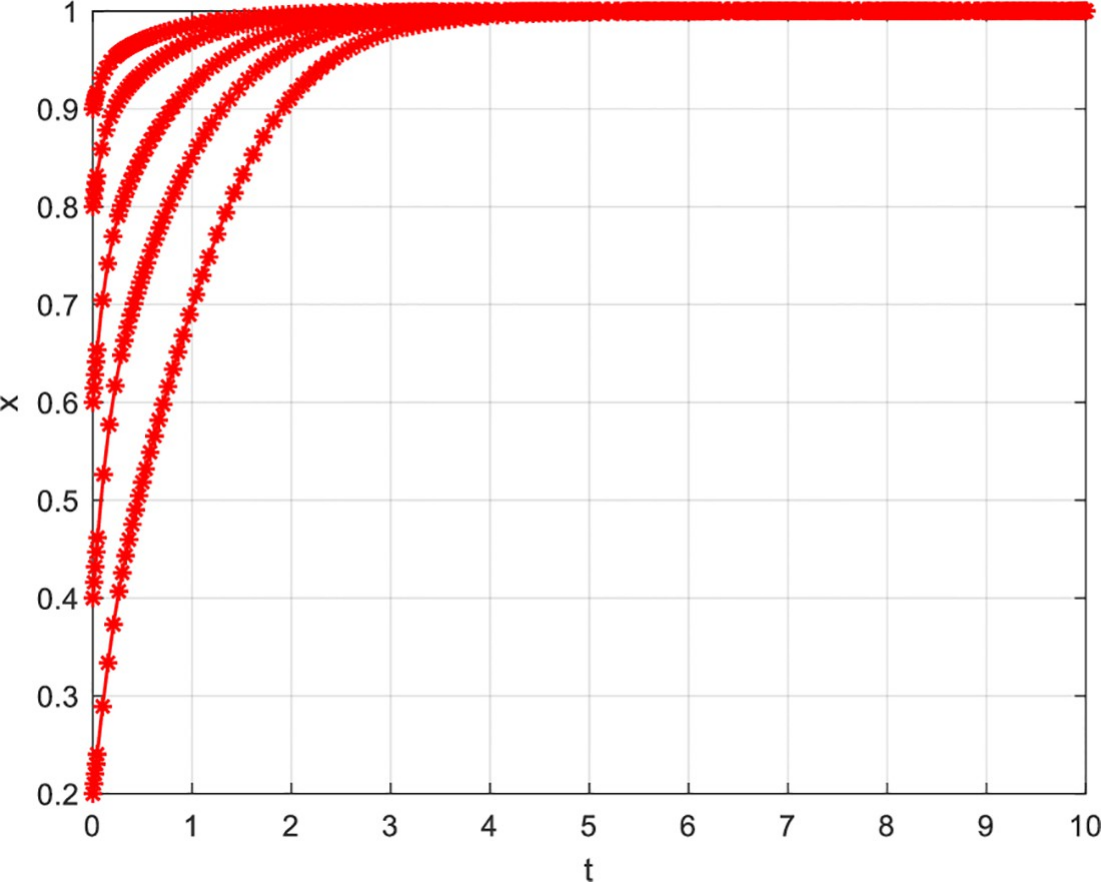


**Fig 7:**
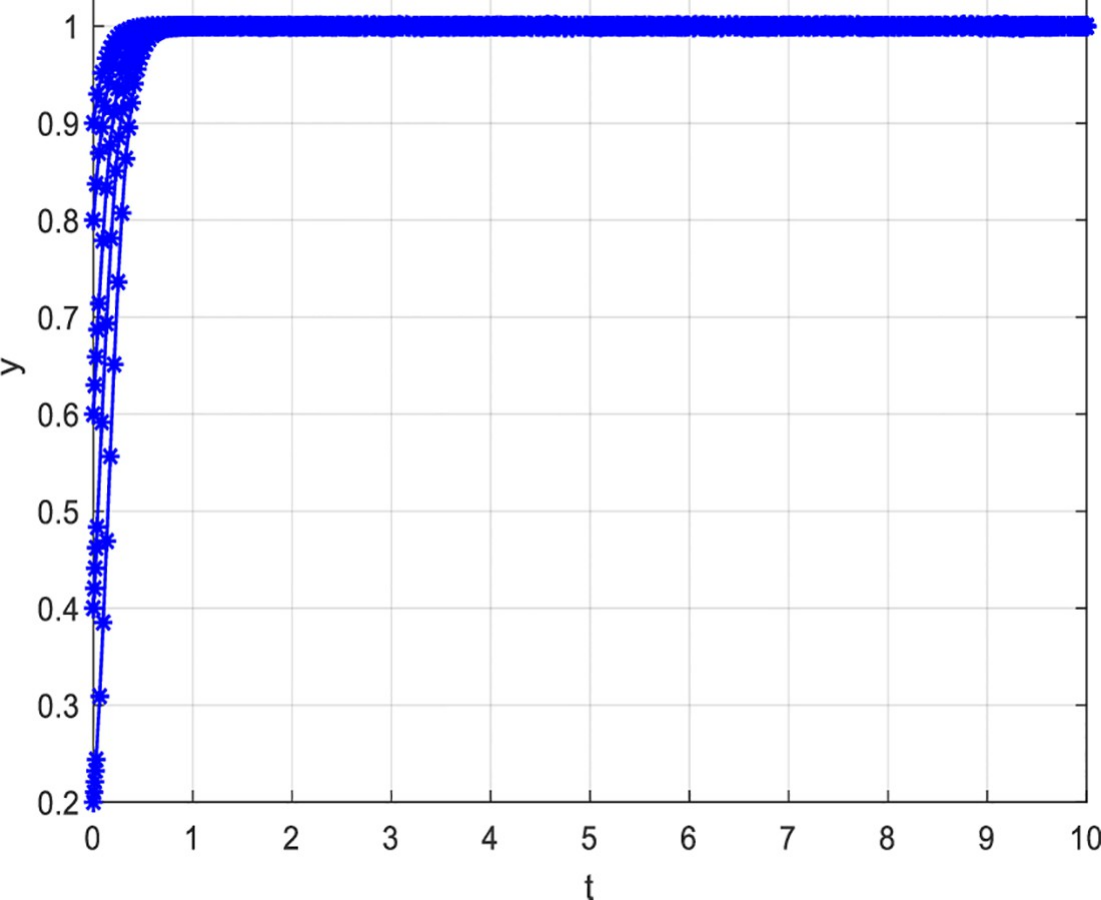


The initial cooperation probability of innovative enterprises influences their willingness to cooperate. Fig 6 illustrates the change in cooperation willingness with an increase in the initial cooperation probability of innovative enterprises while keeping other conditions constant. From Fig 6, it can be observed that, under unchanged conditions, as innovative enterprises’ initial cooperation probability increases, the convergence speed to the equilibrium point also accelerates. Detailed information can be found in Fig 6. Similarly, an increase in the initial cooperation probability of consumers also affects their willingness to cooperate. From Fig 7, it can be seen that, under unchanged conditions, as consumers’ initial cooperation probability increases, the convergence speed to the equilibrium point also accelerates. Detailed information can be found in Fig 7.

Why does an increase in the initial cooperation probability of innovative enterprises and consumers accelerate their convergence to equilibrium? The main reasons are as follows: On one hand, an increase in the initial cooperation probability of innovative enterprises and consumers can enhance the trust and closeness between consumers and enterprises, making it easier for consumers to accept the cooperation proposals and strategies of enterprises, thereby facilitating cooperation and accelerating the pace of coordination. On the other hand, it can also reduce the uncertainty for both parties involved in the cooperation, thereby decreasing the risks and obstacles associated with uncertainty and enabling both parties to adapt to cooperation more quickly, thus speeding up the convergence towards the equilibrium point. Therefore, an increase in the initial cooperation probability of innovative enterprises facilitates their convergence to the equilibrium point.

(2) Variation in loss and Reward Parameters

**Fig 8:**
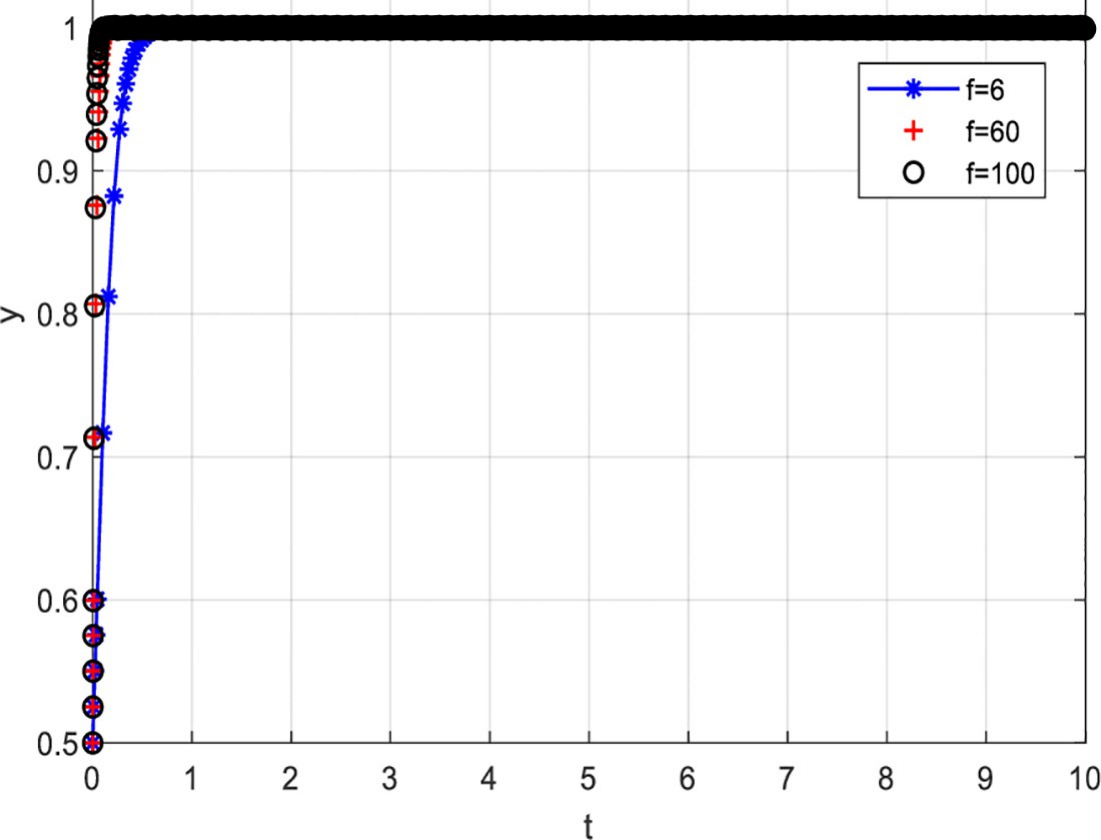


Fig 8 depicts the evolutionary path when consumer losses increase while keeping other conditions constant. From Fig 8, it can be observed that as consumer losses increase, the probability of choosing to participate actively in value co-creation also increases. Specifically, when consumer losses are six units, the probability of consumers participating actively in value co-creation is relatively low. However, as consumer losses increase, i.e., when consumer losses are 60 and 100 units, the probability of consumers actively participating in value co-creation increases. For consumers, bearing certain losses may stimulate their desire to participate actively in value co-creation. When consumers perceive that they may face losses, they will pay more attention to and value the outcomes and development of value co-creation. In order to avoid losses, they will actively participate and contribute their thoughts and opinions. Secondly, the perceived losses ignite a sense of responsibility and mission in consumers, motivating them to participate actively in value co-creation. This sense of responsibility and mission can drive consumers to participate and contribute more actively towards achieving common goals and maximizing benefits. Detailed information can be found in Fig 8.

**Fig 9:**
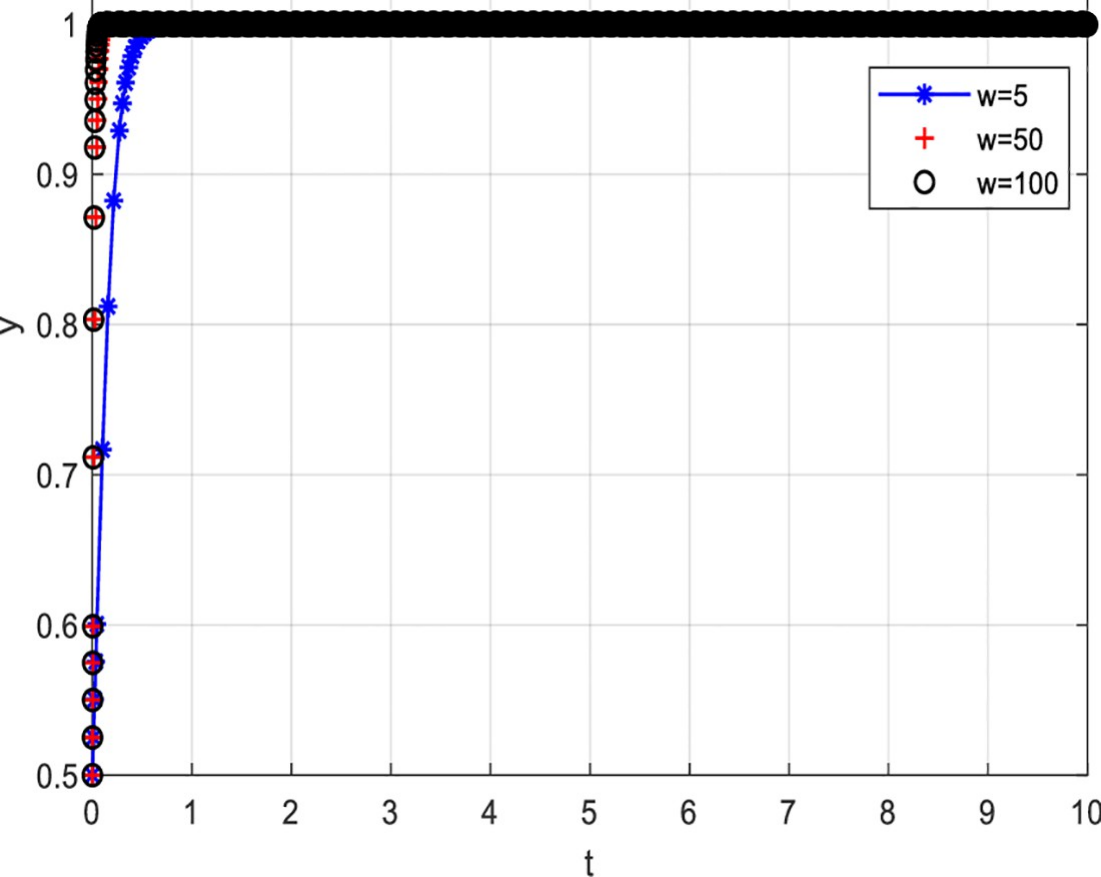

Fig 9 describes the evolutionary path when increasing consumer rewards while keeping other conditions constant. Fig 9 shows that the higher the rewards for consumers, the higher the probability of actively participating in value co-creation. Precisely, when rewards for consumers are five units, consumers’ probability of actively participating in value co-creation is relatively low. However, as rewards for consumers increase, i.e., when rewards are 50 and 100 units, the probability of consumers actively participating in value co-creation increases. The possible reasons are: Firstly, rewards can stimulate consumer interest and motivation. When consumers are rewarded more for value co-creation, they become more motivated to participate actively and willing to invest significant effort and time. Secondly, rewards can enhance the effectiveness and value of consumer participation. When consumers participate in value co-creation, they can contribute by sharing their experiences, ideas, and perspectives to drive and refine the project. Detailed information can be found in Fig 9.

(3) Probability of Being Reported Changes

**Fig 10:**
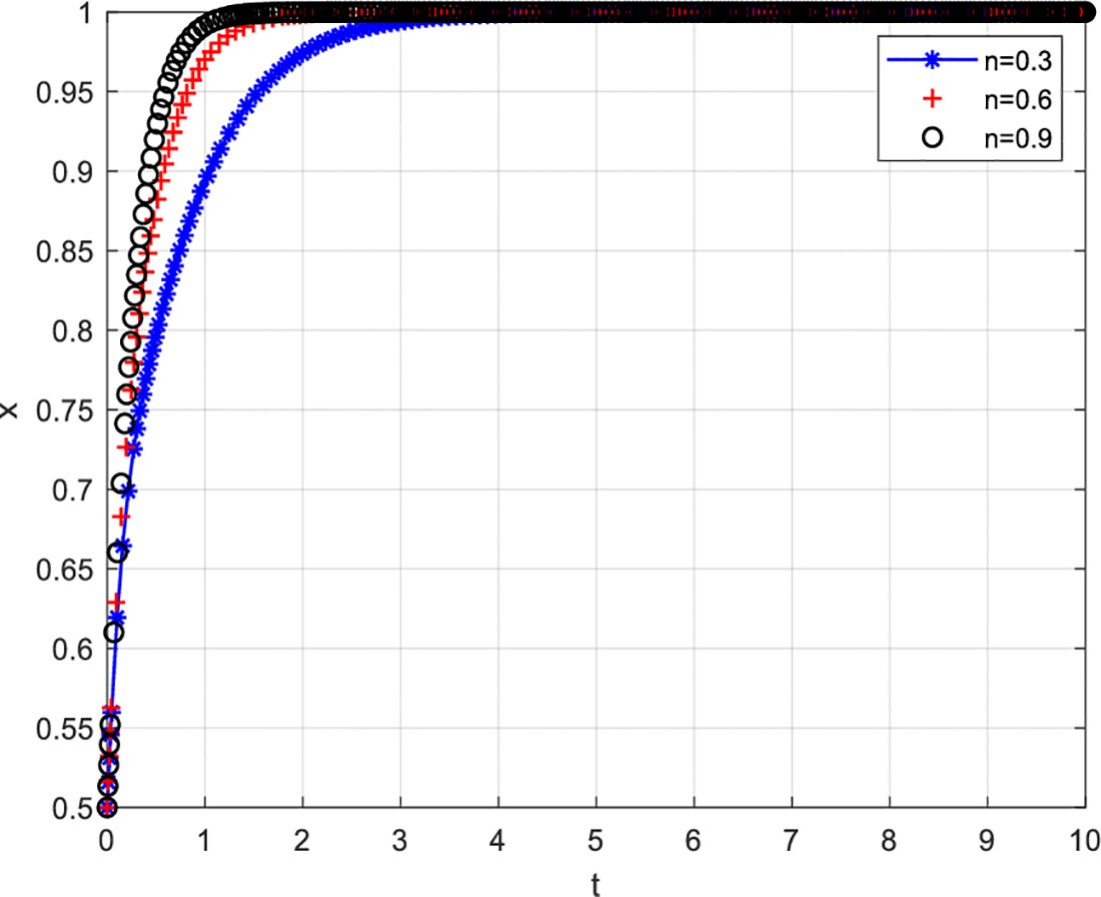


Fig 10 depicts the scenario where innovative enterprises choosing a non-cooperative strategy are reported. Fig 10 shows that the higher the probability of innovative enterprises being reported for choosing a non-cooperative strategy, the higher the probability of them choosing to cooperate. Specifically, when the probability of innovative enterprises being reported is 0.3, the probability of them actively engaging in value co-creation is relatively low. However, as the probability of innovative enterprises being reported increases, i.e., when the probability is 0.6 and 0.9, the probability of them actively engaging in value co-creation also increases. Detailed information can be found in Fig 10.

**Fig 11:**
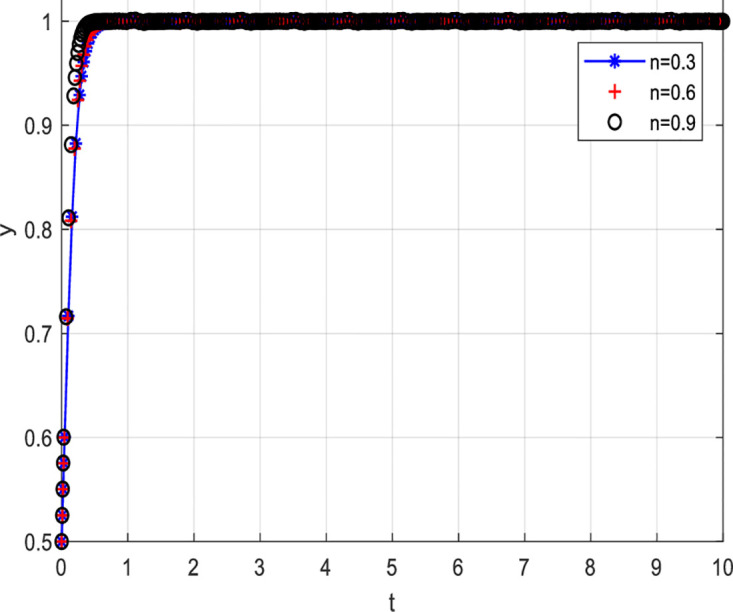


Fig 11 depicts the scenario where consumers choosing a non-cooperative strategy are reported. Fig 11 shows that the higher the probability of consumers being reported for choosing a non-cooperative strategy, the higher the probability of them choosing to cooperate. Specifically, when the probability of consumers being reported is 0.3, the probability of them choosing to cooperate is relatively low. However, as the probability of consumers being reported increases, i.e., when the probability is 0.6 and 0.9, the probability of them choosing to cooperate also increases. Detailed information can be found in Fig 11.

## 6. Conclusion and discussion

Consumers play a significant role in value co-creation with innovative enterprises. A thorough analysis of the mechanisms behind consumer participation in value co-creation with innovative enterprises has essential theoretical and practical implications. Theoretically, studying the mechanisms of consumer participation in value co-creation helps deepen our understanding of consumer needs and values and uncover the influence and impact of consumers on value co-creation within enterprises. This contributes to enriching and expanding consumer value theory, offering essential guidance for enterprise value creation and innovation. Researching the mechanisms of consumer participation in value co-creation with innovative enterprises aligns with the core principles of open innovation theory. It helps reveal the roles and value of consumers in the innovation process, deepening our understanding and application of open innovation theory. By conducting in-depth research on the processes, methods, and effects of consumer participation in innovation, a practical foundation and empirical support can be provided for participatory innovation theories.

From a practical perspective, studying the mechanisms of consumer participation in value co-creation with innovative enterprises can enable businesses to be more market-oriented and better meet consumer demands. Through interaction and collaboration with consumers, enterprises can gain a more accurate understanding of market dynamics and enhance the competitiveness of their products and services. This, in turn, helps to stimulate innovation and entrepreneurial vitality. As innovation’s leading actors and beneficiaries, consumers can provide valuable ideas and feedback, driving better innovation and entrepreneurial practices within enterprises. This can facilitate continuous improvement and optimization of products and services. Through consumer participation and feedback, enterprises can promptly identify and address issues, enhance product and service quality, and improve user experience and satisfaction. The mechanisms of consumer participation in value co-creation with innovative enterprises can also enhance enterprises’ brand image and reputation. Through interaction and collaboration with consumers, enterprises can establish a strong brand image, make improvements and adjustments based on consumer needs and opinions, and enhance brand value and competitiveness.

This study focuses on the game between consumers and innovative enterprises. It provides an in-depth analysis of the underlying mechanisms of consumer participation in value co-creation with innovative enterprises by constructing an evolutionary game model. The following important conclusions are drawn based on the game analysis and systematic simulation analysis. Firstly, the initial cooperation probability between consumers and innovative enterprises directly affects the strategic choices of both parties. Specifically, as the initial cooperation probability between consumers and innovative enterprises increases, both parties choose cooperative strategies. Consumers are more inclined to actively participate in value co-creation strategies, while innovative enterprises are more inclined to engage in value co-creation strategies proactively. Secondly, establishing reward mechanisms makes consumers more inclined to choose to actively participate in value co-creation strategies.

Moreover, as the intensity of rewards increases, the probability of consumers choosing to actively participate in value co-creation strategies also increases. Thirdly, as the probability of both parties being reported for non-cooperation increases, the probability of consumers and innovative enterprises choosing cooperation also increases. In comparison to consumers, the impact of reporting on innovative enterprises is more significant.

Based on the above conclusions, this paper proposes the following strategies and recommendations:

1. Establish a trust relationship and enhance willingness to cooperate. To begin with, a consumer participation platform should be established. On the one hand, enterprises can create dedicated platforms for consumer communication, encouraging consumer involvement in decision-making and innovation activities through online surveys, discussion forums, and idea solicitation, among other methods. On the other hand, enterprises can establish platforms for knowledge and experience sharing, allowing consumers to share and exchange their innovative ideas and experiences. This will help build a learning and innovation community, promoting mutual learning and inspiration among consumers. Secondly, continuously cultivate consumer participation awareness and establish feedback mechanisms. Through relevant promotion and educational activities, enterprises can continuously cultivate consumer awareness and capabilities for innovation. For instance, consumer innovation training courses and relevant innovation tools and methods can be offered to encourage consumers to participate actively in innovation activities. Moreover, enterprises should establish effective feedback mechanisms to collect consumer feedback and opinions promptly. This can be achieved through customer service hotlines, social media interactions, product evaluations, and other means. Valuing and actively responding to consumer feedback will help enterprises improve their products and services, enhancing their competitiveness and customer satisfaction.
2. Establish reward and incentive mechanisms and actively engage in cooperation. Establishing reward and incentive mechanisms to encourage consumer participation in value co-creation with innovative enterprises is essential. For example, consumer innovation awards can be established to provide recognition and rewards to consumers who contribute significantly, stimulating more consumer involvement in innovation activities. Additionally, enterprises can collaborate with consumers on research and development projects, involving them in product design and feature development activities. Through collaborative R&D with consumers, enterprises can better understand consumer needs and enhance the market adaptability and competitiveness of their products.
3. Establish a reasonable reporting mechanism to urge consumers to co-create value with innovative enterprises. Firstly, strengthen monitoring and inspection of non-cooperative behavior, such as increasing the frequency and coverage of regulatory inspections to increase the likelihood of detecting non-cooperative behavior. Secondly, establish reward and punishment mechanisms. For example, rewarding individuals who discover and report non-cooperative behavior while simultaneously imposing penalties on reported non-cooperative behavior to increase the likelihood of being reported. An anonymous reporting mechanism can also be established to allow individuals unwilling to report formally to report anonymously, thereby increasing the likelihood of reporting non-cooperative behavior.

This paper focuses on the game between consumers and innovative enterprises. It establishes a bi-directional evolutionary game model to analyze the mechanisms of consumer participation in value co-creation with innovative enterprises. However, there are some limitations in exploring consumer participation in value co-creation with innovative enterprises. Firstly, the role of government in value co-creation with innovative enterprises is not considered. The government plays a crucial role in value co-creation with innovative enterprises by providing policy support, tax incentives, and other forms of assistance. Secondly, repeated games need to be considered when discussing consumer participation in value co-creation with innovative enterprises. Consumer participation in value co-creation with innovative enterprises is a progressive and repetitive game process. Therefore, in future research, the government could be included in the game model, and the issue of repeated games should be considered in analyzing the game process.

## Supporting information

S1 File Auxiliary data files. (DOCX)
