# Supplementary material for: Research on the mechanism of consumer participation in value co-creation by innovative enterprises: An evolutionary game analysis framework
Source: PLoS One. 2024 May 15;19(5):e0297475. doi: 10.1371/journal.pone.0297475 (PMC11095749; doi:10.1371/journal.pone.0297475)
Supplement: S1 File — (DOCX) [file pone.0297475.s001.docx]

Table . Parameter settings for Scenarios 1-4

| Parameter | $c_{E1}$ | $c_{E2}$ | $R_{E1}$ | $c_{C}$ | $\omega$ | $f$ | $\eta$ | $R_{E}$ | $R_{C}$ |
| --- | --- | --- | --- | --- | --- | --- | --- | --- | --- |
| Scenario 1 | 5 | 10 | 10 | 4 | 5 | 6 | 0.3 | 5 | 5 |
| Scenario 2 | 7 | 10 | 10 | 4 | 5 | 6 | 0.3 | 5 | 5 |
| Scenario 3 | 3 | 4 | 3 | 4 | 5 | 6 | 0.7 | 10 | 5 |
| Scenario 4 | 5 | 4 | 3 | 4 | 5 | 6 | 0.7 | 5 | 5 |
